# Steel work design and analysis of a 40-ton constant temperature hydraulic press

**DOI:** 10.1016/j.heliyon.2020.e04783

**Published:** 2020-09-03

**Authors:** Paul Chukwulozie Okolie, Echezona Nnaemeka Obika, Benjamin Segun Oluwadare, Obiora Nnaemeka Ezenwa, Chinedu Stanley Udensi

**Affiliations:** a Mechanical Engineering Department, Nnamdi Azikiwe University, PMB 5025, Awka, Anambra State, Nigeria; b Mechanical Engineering Department, Ekiti State University, PMB 5363, Ado Ekiti, Ekiti State, Nigeria

**Keywords:** Industrial engineering, Materials science, Mechanical engineering, Load, Compression, Motorized, Mobility, Pressure, Spring

## Abstract

This study presented the analyses of the design and construction of a motorized 40-ton constant temperature hydraulic press, for constant temperature compression purposes of fibre matrices. Standard design considerations and calculations were done to ensure the selection of effective and efficient components for the development of the machine. The construction of the machine involved standard manufacturing processes which involved marking out, cutting, drilling, machining and welding processes. The design was motorized for the purpose of increasing mechanical advantage as against hand-powered press. Design analyses were carried out to facilitate accurate dimensioning of the various parts of the hydraulic press. For adequate spring selection, the spring analysis of the system was also done. On testing of the finished device, no sign of leakages and system failure were observed. The effect of press time, temperature and pressure on the density of test samples, during a manufacturing process using the developed machine showed good working condition on both the compression and heating processes of the machine.

## 1 Introduction

Hot pressing is a metallurgical process that involves high-pressure, low-strain-rate for forming of a powder compact at a high temperature capable of inducing sintering and creep processes. Hot pressing is achieved with a hot hydraulic press (hydraulic press equipped with a heater). While the hydraulic press is responsible for the compression operation by exerting high compressive force (Tejas et al., 2015), the heating device supplies the needed heat for the heating operation. The heat supply can either be constant or varied using a temperature controller for the purpose melting materials into shapes or altering their structure based on their uses and requirement.

In workshops and laboratories, a hydraulic press is an essential equipment for press-fitting operations and material deformation purposes such as metal forming processes and strength of material test (Sumaila and Ibhadode, 2011). In a hydraulic press, the much needed compressive force is generated using the hydraulic cylinder arrangement (Kumar and Prashanth, 2017). The hydraulic press is used for different mechanical processes such as installation and removal of bearings (Nwankwojike et al., 2017) and strength of material test (Dagwa and Ighadode, 2005). According to Parker and Dana (2013) the hydraulic press was invented by Joseph Bramah in England in 1795, in accordance with the Pascal's principle of constant pressure throughout a closed system. Works on hydraulic press include that of Ashwin Joshi (2015), a 5 tonnes hydraulic press, having a working pressure of 150bar in a double acting cylinder arrangement and H-frame type construction, with a power pack unit. Also, Sumaila and Ibhadode (2011) designed and constructed a 30-ton hydraulic press using locally sourced material with a piston stroke of 150 mm. Muni and Amarnath (2011) optimized the structure of a 40 Ton Hydraulic Press and Scrap Baling Press for the purpose of cost reduction by applying suitable loads and constraints to the initial design space of the components. Mohammed et al (2020) designed the press frame, cylinder and press table of a hydraulic press. They aimed at cost reduction through mass minimization of an H frame type hydraulic press. For laboratory purposes, Amiolemhen and Ogie (2019) developed a 10-ton hydraulic press putting into consideration the load resistance distance, system pressure, cylinder area and the volume flow rate of the working fluid. To promote mechanised farming, Adinife et al. (2013) designed a motorized hydraulically operated palm oil press. They achieved a machine capacity of 330.96 KN/m^2^ and an average pressing time of 4.35mins. Lackner (2004) noted that for the purposes of compacting irregular geometries and prompt reaction to production requirements, innovative electronic and hydraulic components should be put to use.

Stishov (2019) designed a laboratory hydraulic press with a force of 20 tons, with its special feature being the use of a spring for reversing the piston, thus simplifying the operating procedure. Ravindra et al. (2019) constructed a manually operated 5ton hydraulic press to increase mechanical advantage in domestic oil extraction. The 30-ton hydraulic forming press machine design of Bapat and Desai (2016) presented the structural analysis of the device as well as its optimisation to prevent structural failure. The study of Parthiban et al., (2014) saw the design and analysis of C-type hydraulic press for enhanced rigidity, noting that structural behaviour is dependent on the shape of the frame.

Thus this research is aimed at the development of a low cost constant pressure hydraulic press that will facilitate research especially in the area of material science and production in our universities.

## 2 Materials and method

### 2.1 Material preference

Materials used in this work were chosen according to design standards, putting into consideration various factors (Okolie et al., 2019). These factors include; cost, strength, machinability, availability, etc (Chukwulozie et al., 2016). The selected materials and the reason for their selection are summarised in Table 1.

**Table 1:** Chosen materials for the components of the press.

S/N	Component	Chosen Material	Considered factors
1	Columns	Mild steel	Cost, machinability, high strength in tension and availability
2	Platen plate and base plate	High carbon steel	High strength in compression and high thermal conductivity
3	Spring	Oil tempered steel	High strength in tension and rigidity
4	Nuts	High carbon steel	High strength in tension and shear
5	Tires	Steel	High compressive strength

### 2.2 Description of the components of the press machine

The various parts of the device are;

1) **Hydraulic Cylinder**: This provides the needed pressure required for the pressing operation of the device.
2) **Columns**: They are the vertical metallic members that support the platens (upper and lower high carbon steel plates) for pressing operations. The columns are fitted through the platens (passes through holes made on the platen), to allow the lower platen to slide freely through it.
3) **Platens or Ram plates:** These are the two heated high carbon steel plates with an inner extruded plate. The pressing and heating operation is done in between them.
4) **Motor:** This device converts electrical energy into mechanical energy needed for the actuation of the lift cylinder.
5) **Springs:** These components are elastic and are used to return the lower platen to its original position after the hot pressing operation has been carried out. The springs are made of oil-tempered steel to withstand the tension force as the hydraulic jack piston pushes the lower platen upward.
6) **Hydraulic pump:** This component creates the vacuum needed to force the movement of the hydraulic fluid from the reservoir to the hydraulic jack supply port.
7) **Pressure gauge:** The pressure gauge indicates the pressure value as the hydraulic jack piston is being raised by the action of the electric motor.
8) **Temperature control device:** The temperature control device supplies a variable current from 4000 W to the platen plates and they are two in number.
9) **Hoses:** These are hollow tubes with thickness needed to either supply or return hydraulic fluid to the lift cylinder or reservoir respectively.
10) **Control valves:** These valves control the flow of fluid between the lift cylinder and reservoir.
11) **Fluid reservoir:** This is the storage tank for the hydraulic fluid.
12) **Rollers**: These provide mobility to the device.
13) **Base plate**: This houses all other components of the press machine. The base plate is fitted with tires to aid mobility.

### 2.3 Construction

**Figure 1:**
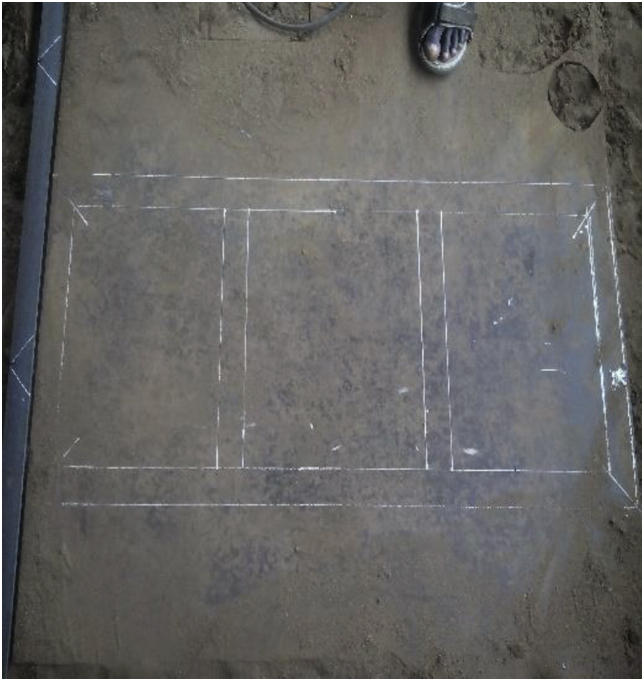
Marking out operation.

At this stage, work is done on the different parts that form the system to bring them to a suitable shape and size required for the design. The process for fabrication includes;

a) Marking out the operation: This operation involved setting off dimensions and punching out points on the metal used for the platens, columns, base plate using the punch tool as seen in Figure 1.

**Figure 2:**
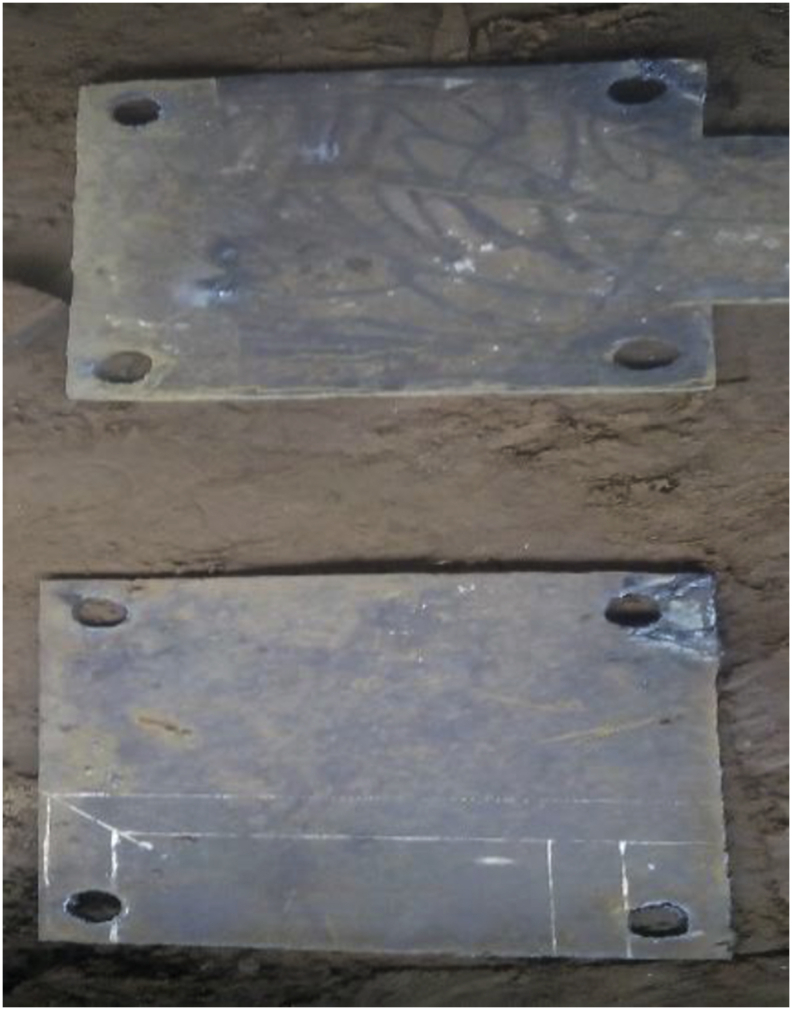
Cutting and drilling operation.b)Cutting operation: it includes cutting out the required size and shapes for the base plate, columns and platens. This operation was done employing both manual and electric powered saw.c)Drilling operation: In this operation, the required holes were drilled on the components using the drilling machine to produce the shape shown in Figure 2.d)**Assembling**: This process consists of putting together of all individual elements or parts that have been worked on to form the system. This stage involves the welding operation, screwing operation and finishing operations. On completing the assembly operation Figure 3 will be obtained.

**Figure 3:**
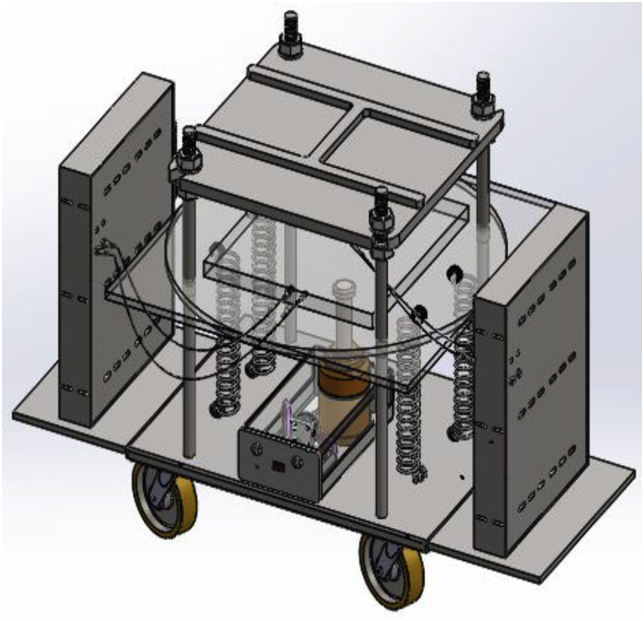
Motorized 40-ton hydraulic hot press machine.

## 3 Design analysis

### 3.1 Design for press plate

A square plate of length 0.3m was used for the press surface. To determine the thickness ‘t’ of the plate, the compressive strength of the plate material, the maximum force acting on the plate as well as the area of the surface will be considered using the expression;

(1) σ=3FL2bt2(Rajput,2010)

where;

σ= Compressive strength of mild steel $=320N/m^{2}$ (Rajput,2010)

F = force exerted on plate

L = length of plate

b = width of the plate

t = thickness of the plate

Since the plate is square in shape the area ‘A’ becomes;

(2) $$A=L^{2}$$

Hence, A = 0.09m.

The desired maximum press pressure for the hot press ‘P’ is 25bar.

To calculate the required force we make use of Eq. (3);

(3) $$P=\frac{F_{p}}{A}$$

Thus the required force becomes; $F_{p}=225KN$

Solving for the thickness of the plate using Eq. (1) we have

t=3FpL2σb

Thus t = 0.031m.

### 3.2 Design for hydraulic cylinder

**Figure 4:**
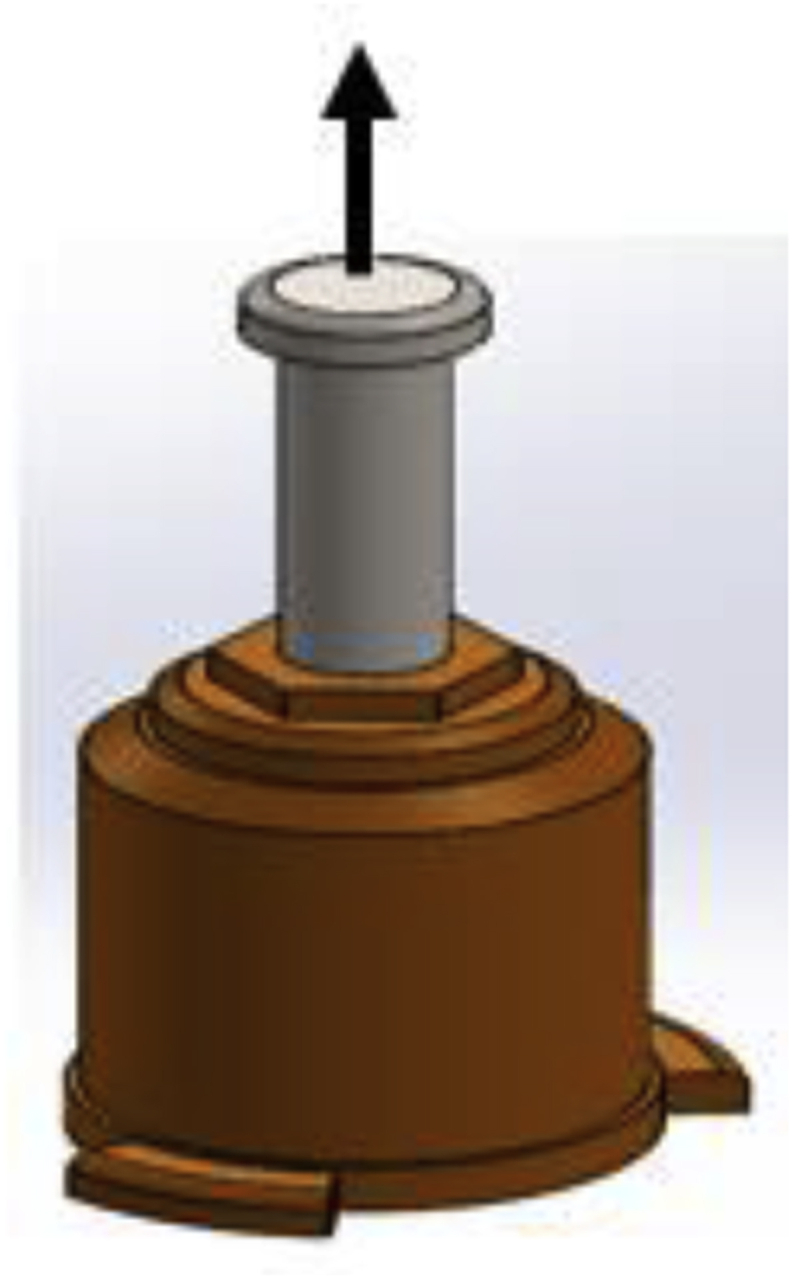
Free body diagram of hydraulic cylinder.

For proper selection of the required capacity of hydraulic cylinder the total force ‘F’ (with the direction described by the arrow in Figure 4) which is the sum of the required press force F_p_ and weight of the plate ‘W’, was determined using Eq. (4);

(4) $$F=F_{p}+W$$

But;

(5) W=mg

where; m = mass of plate and g = acceleration due to gravity.

With $density\;of\;mild\;steel=7850kg/m^{3}$ and volume of plate = 0.3×0.3×0.031=0.00279, the total force force ‘F’ becomes;

F=225219N

Considering a factor of safety of 1.5, the total force therefore becomes;

225219×1.5=337828.5N=38tons

Hence a hydraulic cylinder of 40tons was selected.

### 3.3 Design of base and upper plate

The force exerted on the base is 337828.5N (38tons).

The total surface area of base plate is given as;

$$A=0.5m\times 0.5m=0.25m^{2}$$

Applying Eq. (1) the thickness of base plate becomes;

$$t_{b}=0.025m$$

Since action and reaction are equal and opposite, the force of 337828.5N will be applied to the upper plate surface area of 0.3×0.3m, hence using Eq. (2) the thickness of the upper plate will be;

$$t_{u}=0.025m$$

### 3.4 Design for columns

**Figure 5:**
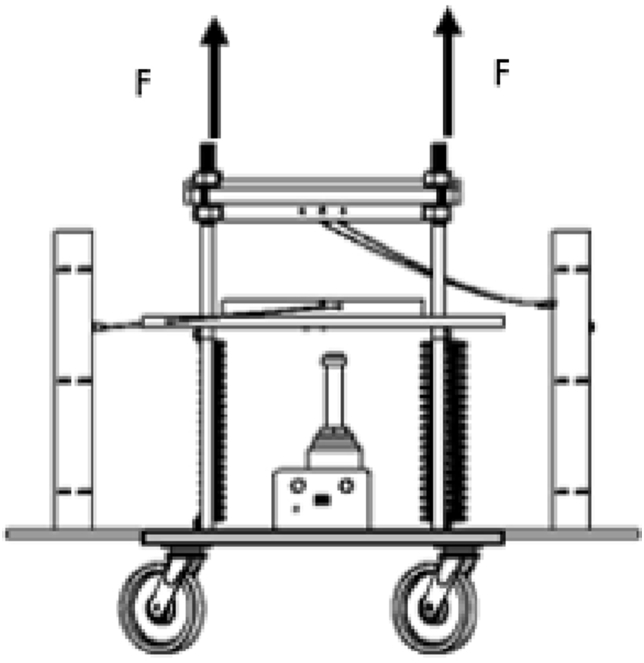
Free body diagram showing force acting on column.

Since there are four columns, the force (as seen in Figure 5) acting on each column is $\frac{337828.5}{4}=84457.125N$.

Area of circular face of column = $\pi \frac{d^{2}}{4}$

Tensile strength of mild steel bar = 400MPa.

Combining the formula $\sigma =\frac{F}{A}$ and area of circle surface the diameter of column becomes;

(6) $$d=\sqrt{\frac{4F}{\pi \sigma }}$$

d=16mm

### 3.5 Design for electric motor shaft

Formulas for the design of motor shafts are:

(7) $$T=\frac{60P}{2\pi N}$$

P = power transmitted by a 1hp, single phase motor which is 746watts.

N = speed associated with a 1hp, single phase motor which is 1400rpm

$$T=\;\frac{60\times 746}{2\times \pi \times 1400}=5.09Nm$$

Measured mass of the shaft, M = 21.3g.

From the theory of maximum shear stress (Guest or Tresca's theory), the diameter, ‘d’ of the shaft is given as:

(8) $$\text{d}={\left(\frac{16}{\pi \tau }\sqrt{{\left(K_{B}M_{B}\right)}^{2}+{\left(K_{T}M_{T}\right)}^{2}}\right)}^{\frac{1}{3}}$$

where;

$K_{B}=$Bending moment factor

$K_{T}=$Torsional moment factor

$M_{B}=$Bending moment associated with the weight of crank and connecting rod

$M_{T}=$Torque or torsional moment

Shear stress,

(9) $$\tau =\frac{Ss_{y}}{n}=\frac{Sy}{2n}$$

Since no load acts on the shaft, M_b_ = 0 and taking K_t_ = 1, the diameter now becomes;

(10) $$d={\left(\frac{16\times M_{t}}{\pi \tau }\right)}^{\frac{1}{3}}$$

Taking the shear stress, τ = 87.5 Mpa for a steel shaft

$$d={\left(\frac{16\times 5.09}{\pi \times 87.5\times 10^{6}}\right)}^{\frac{1}{3}}=6.66mm$$

d = 6.66mm

### 3.6 Design for motor

The motor chosen was to provide high torque at low speed to avoid vibration of the system during operation. The design for motor output power is vital for appropriate motor selection.

(11) P=Txw

where;

P = power (watts)

T = rotational force (torque) acting on the shaft in Newton (N)

w = angular velocity of the shaft (rad/s)

But also Torque,

(12) $$T\;=\;I\bar{\alpha }$$

I = moment of inertia of rotating shaft (kgm^2^)

ᾱ = angular acceleration of the motor in radian/seconds square (rad/s^2^)

(13) $$\bar{\alpha }\;=\;w²r$$

r = radius of the motor shaft (m)

Also

(14) w=v/r

Therefore: v = wr.where:

v = linear velocity (m/s)

By putting Eq. (13) into (12)

(15) $$\text{T }=\;{\text{Iw}}^{2}\text{r}$$

By putting Eqs. (14) and (15) into (12)

(16) $$P=Iw^{2}r\times \frac{v}{r}$$

But

(17) $$w=\frac{2\pi N}{60}$$

Thus;

(18) $$P=\frac{2\pi NT}{60}$$

But for our design, the power required for the jack to be raised could be determined using Eq. (19)

(19) $$P\;=\;F_{2}\;x\;v$$

where

F_2_ = force required to press smaller piston (N)

v = velocity of fluid to provide the necessary pressure (m/s)

From the Pascal's law, to provide a 40-ton pressing force (392,400N).

(20) $$\frac{F_{1}}{A_{1}}=\frac{F_{2}}{A_{2}}\;\text{i.e.}\;\frac{F_{1}}{{d_{1}}^{2}}=\frac{F_{2}}{{d_{2}}^{2}}$$

F_1_ = output force of the jack = 392,400N

d_1_ = diameter of hydraulic piston = 50mm

d_2_ = diameter of smaller piston = 10mm

$$F_{2}=\frac{F_{1}\times {d_{2}}^{2}}{{d_{1}}^{2}}=\frac{392,400\times 10^{2}}{50^{2}}=15,696N$$

For a pump mean flow rate of 0.217 L/min (3.613 × 10^−6^ m^3^/s)

Hose diameter, d of 0.01m.

Fluid velocity,$v=\frac{Q\left(m^{3}/s\right)}{A\left(m^{2}\right)}$= Flow rate/Hose Area

$$v=\frac{3.613\times 10^{-6}}{\left[\frac{\pi \times 0.01^{2}}{4}\right]}=0.046m/s$$

$$Power\;input\;=\;F_{2}\;x\;v\;=\;15,696\;x\;0.046\;=\;723.05\;watts\;=\;0.97hp$$

Therefore, we select a 1hp motor.

### 3.7 Design for springs

The springs employed in this design are oil-tempered steel under a tensile load of 392400N from the hydraulic jack.

W = 392400N

**Figure 6:**
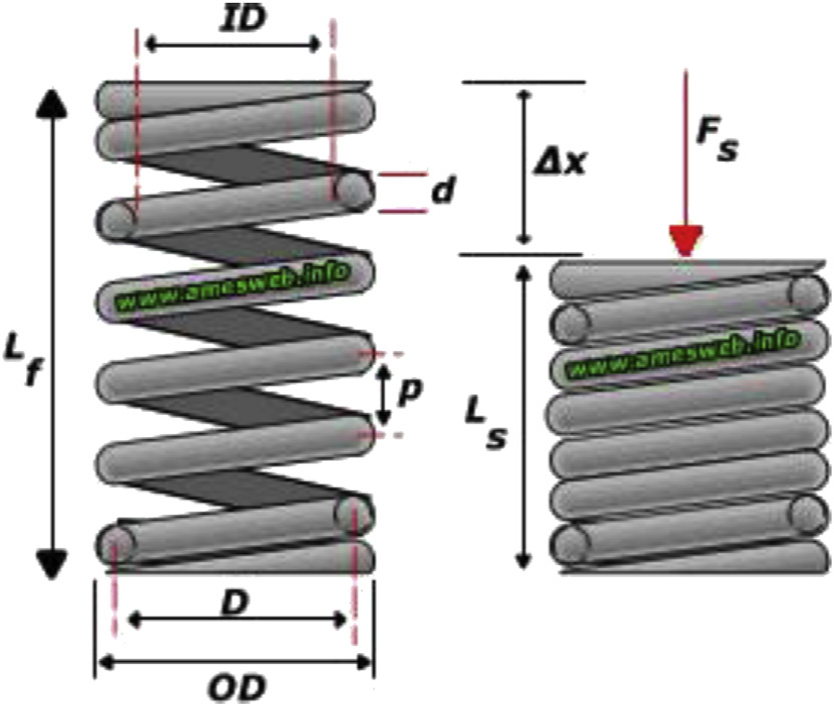
Free body diagram of spring.

The free body diagram of the spring is shown in Figure 6. The total number of coils, n’, on the spring is 44.

Solid length (when compressed),

(21) $$L_{S}=n^{'}.d_{sp}$$

Free Length (unloaded condition),

(22) $$\;L_{f}=n^{'}.d_{sp}+\delta_{max}+0.15\delta_{max}$$

Spring index,

(23) $$C=\frac{D}{d_{sp}}$$

Spring Rate/Spring Constant,

(24) $$k=\frac{W}{\delta }$$

Pitch of the spring,

(25) $$P=\frac{L_{f}}{n^{,}-1}=\frac{L_{f}-L_{s}}{n}+d_{sp}$$

where

$n^{'}$ = Total number of coils

d_sp_ = wire's diameter

δ_max_ = maximum compression

D = coil's mean diameter

δ = spring's deflection

k = spring constant

And given as associated with an oil-tempered spring:

n’ = 44, d_sp_ = 1.5mm, δ = 18.5mm, D = 12.5mm, k = 21211N/mm, C = 8.33.

Therefore;

Solid length, L_S_ = 44 × 1.5mm = 66mm

Free length, L_f_ = 44 × 1.5mm + 18.5mm + 0.15 × 18.5mm = 87.275mm

Pitch, p = $\frac{87.275}{44-1}=2.03mm$

For spring pitch selection, the following factors are to be considered.

• The pitch of the spring should be designed such that the stress does not exceed the yield point torsion stress in a case of accidental compression of the spring.
• Ensure that the spring is not closed until the maximum service load is reached (Khurmi and Gupta, 2005).

### 3.8 Stresses in helical springs of circular wire

By considering a helical spring with load (W), an application of load W tends to the rotation of the wire due to twisting moment (T) set up in the wire. Furthermore, torsional stress that is produced in the wire due to twisting can be calculated using Eq. (26).

(26) $$\tau_{1}=\frac{8W.D}{\pi d^{3}}=\frac{8\times 392400N\times 12.5mm}{\pi \times {\left(1.5mm\right)}^{3}}=3700.9kN/mm^{2}$$

In addition to the torsional shear stress (τ_1_) that is produced in the wire, the stresses below also act it:

• Direct shear stress due to the load, W

(27) $$\tau_{2}=\frac{4W}{\pi d^{2}}=\frac{4\times 392400N}{\pi \times {\left(1.5mm\right)}^{2}}=222.05kN/mm^{2}$$

• Stress due to curvature of wire (Khurmi and Gupta, 2005).

Resultant shear stress induced in the wire is given as:

(28) $$\tau =\tau_{1}+\tau_{2}=\frac{8W.D}{\pi d^{3}}+\frac{4W}{\pi d^{2}}==3922.95kN/mm^{2}$$

Maximum shear stress induced in the wire = Resultant shear stress induced in the wire.

Also, maximum shear stress produced in the wire can be given as:

(29) $$\tau s{{\frac{8W.D}{\pi d^{3}}}^{2}}_{max}$$

where

(30) $${\text{K}}_{\text{s}}=\text{ shear}\;\text{stress}\;\text{factor }=\;1+\frac{1}{2C}=1.06$$

To consider the effect of curvature of wire, we introduce a Wahl's stress factor (K), therefore

(31) $$\tau \frac{8W.D}{\pi d^{3}}{{\frac{8W.C}{\pi d^{2}}}^{2}}_{max}$$

where

(32) $$K=\frac{4C-1}{4C-4}+\frac{0.615}{C}=1.176$$

The Wahl's stress factor (K) consists of two sub factors, K_s_ and K_c_, such that

(33) $$\text{K }=\;{\text{K}}_{\text{s}}\text{ x }{\text{K}}_{\text{c}}$$

where

K_s_ = Stress factor due to shear, and

K_c_ = Stress concentration factor due to curvature.

### 3.9 Spring-mass analysis of the system

**Figure 7:**
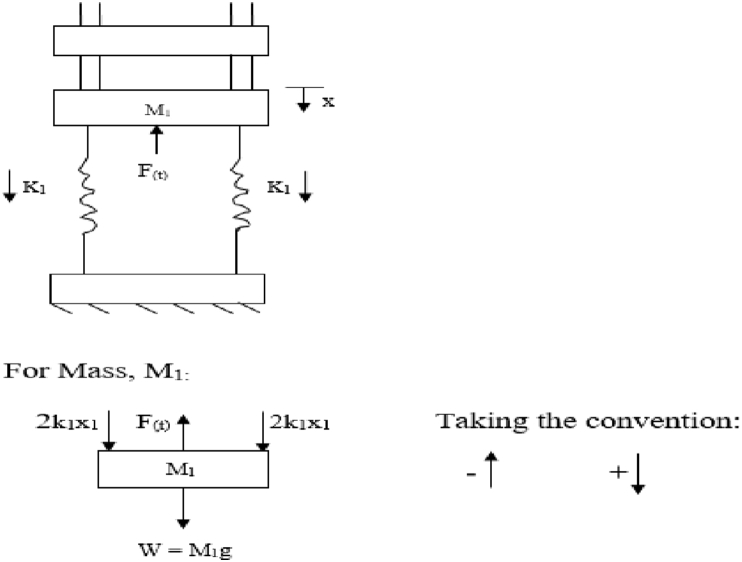
Free body diagram for spring-mass analysis.

From the hydraulic press system, the free body diagram can be drawn as shown in Figure 7;

Considering four springs, two on each side. Therefore, summing the upward and downward forces:

Elemental equation:

(34) $$M_{1}\frac{d^{2}x_{1}}{dt^{2}}+2k_{1}x_{1}+2k_{1}x_{1}=F\left(t\right)$$

Taking the Laplace transform of the elements:

(35) $$M_{1}\left[s^{2}x_{1}\left(s\right)-sx_{1}\left(0\right)-{x_{1}}^{1}\left(0\right)\right]+2k_{1}x_{1}\left(s\right)+2k_{1}x_{1}\left(s\right)=F\left(s\right)$$

From initial conditions, At $x_{1}\left(0\right)$ = 0 and ${x_{1}}^{1}\left(0\right)$ = 0

(36) $$\begin{array}{l} M_{1}s^{2}x_{1}\left(s\right)+2k_{1}x_{1}\left(s\right)+2k_{1}x_{1}\left(s\right)=F\left(s\right)\\ x_{1}\left(s\right)\left[M_{1}s^{2}+2k_{1}+2k_{1}\right]=F\left(s\right) \end{array}$$

Therefore, the transfer function of the system becomes;

(37) $$\frac{x_{1}\left(s\right)}{F\left(s\right)}=\frac{1}{M_{1}s^{2}+2k_{1}+2k_{1}}=\frac{1}{M_{1}s^{2}+4k_{1}}$$

Therefore given spring constant, k = 21211N/mm

Measured mass of the platen plate, M_1_ = 2.4kg

Maximum deflection, X_1_ = 18.5mm

Input force, F = 15,696N

Substituting into Eq. (34) gives the transfer function factor as

$$\frac{x_{1}\left(s\right)}{F\left(s\right)}=0.00118.$$

## 4 Result and discussion

The testing of engineering products to ascertain the level of performance is an expedient step that needs to be considered during manufacturing processes (Sumaila and Ibhadode, 2011). The design Expert software was used to set up experiment in order to ascertain the performance of the machine as well as to determine the effect of press time, temperature and pressure on the density of test samples. The machine was utilized for the production of ceiling board using recycled low density polyethylene (rLDPE) and breadfruit seed coat. These materials were used due to their good thermal and mechanical properties.

Table 2, shows the experimental factors and response that were considered in the test evaluation of the machine. The factors considered are press time, temperature and pressure, while the response is density.

**Table 2:** Experimental factors and Response.

S/N	A:TIME (Mins)	B:TEMPERATURE (^o^C)	C:PRESSURE (Bar)	DENSITY (Kg/cm^3^)
1	2	120	5	800
2	5	120	5	531
3	2	180	5	750
4	5	180	5	570
5	2	120	10	780
6	5	120	10	612
7	2	180	10	700
8	5	180	10	510
9	1	150	7.5	750
10	6	150	7.5	622
11	3.5	100	7.5	700
12	3.5	200	7.5	370
13	3.5	150	3.3	760
14	3.5	150	11.7	730
15	3.5	150	7.5	600
16	3.5	150	7.5	630
17	3.5	150	7.5	650
18	3.5	150	7.5	630
19	3.5	150	7.5	700
20	3.5	150	7.5	630

### 4.1 3D surface plot

The 3D surface plot gives the combined effect of factors on a particular response. It shows the response surface estimation as a function of two factors with all other factors held constant. With the 3D response surface plot, the effect of the interaction of two factors on a particular response could be studied.

**Figure 8:**
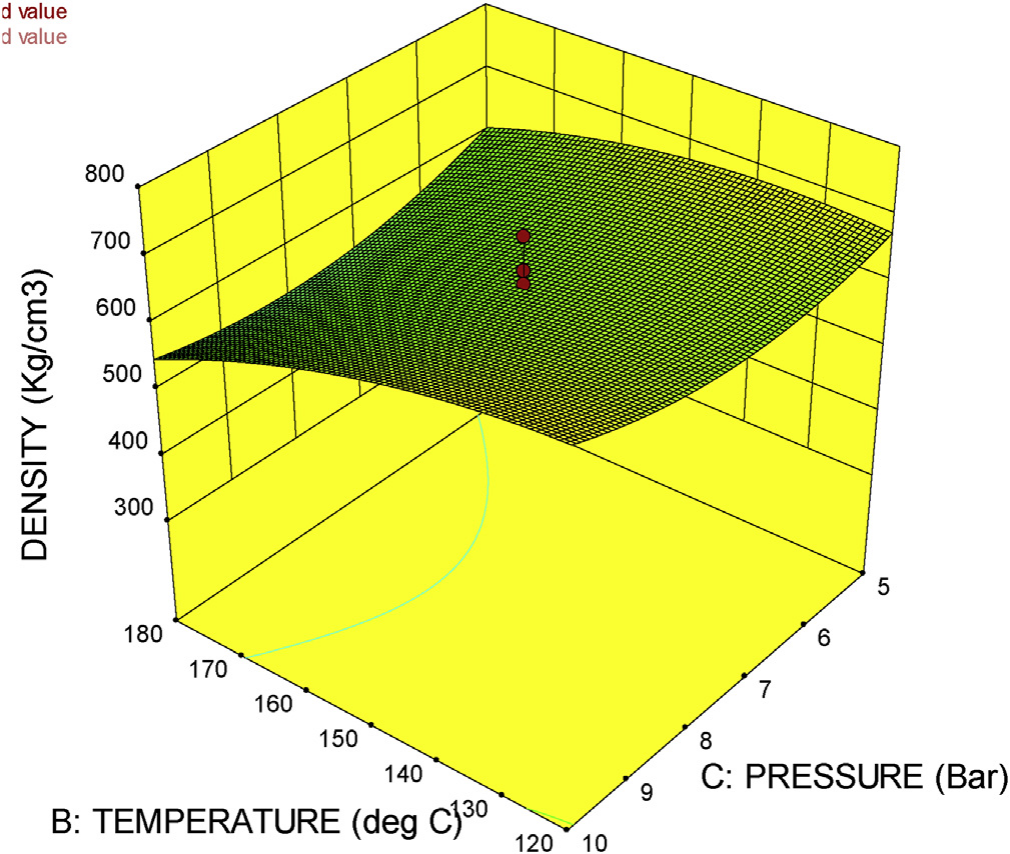
3D surface plot for press temperature against press pressure.

Figure 8, shows the 3D surface plot for press temperature against press pressure. It could be seen from the figure that the increase in temperature and pressure reduces the density of the material. This is justified with the fact that increase in temperature increases evaporation which in turn reduces the moisture content of the material. The increase in pressure on the other hand also reduces the moisture content of the material. This findings agree with that of Ezenwa et al. (2019).

**Figure 9:**
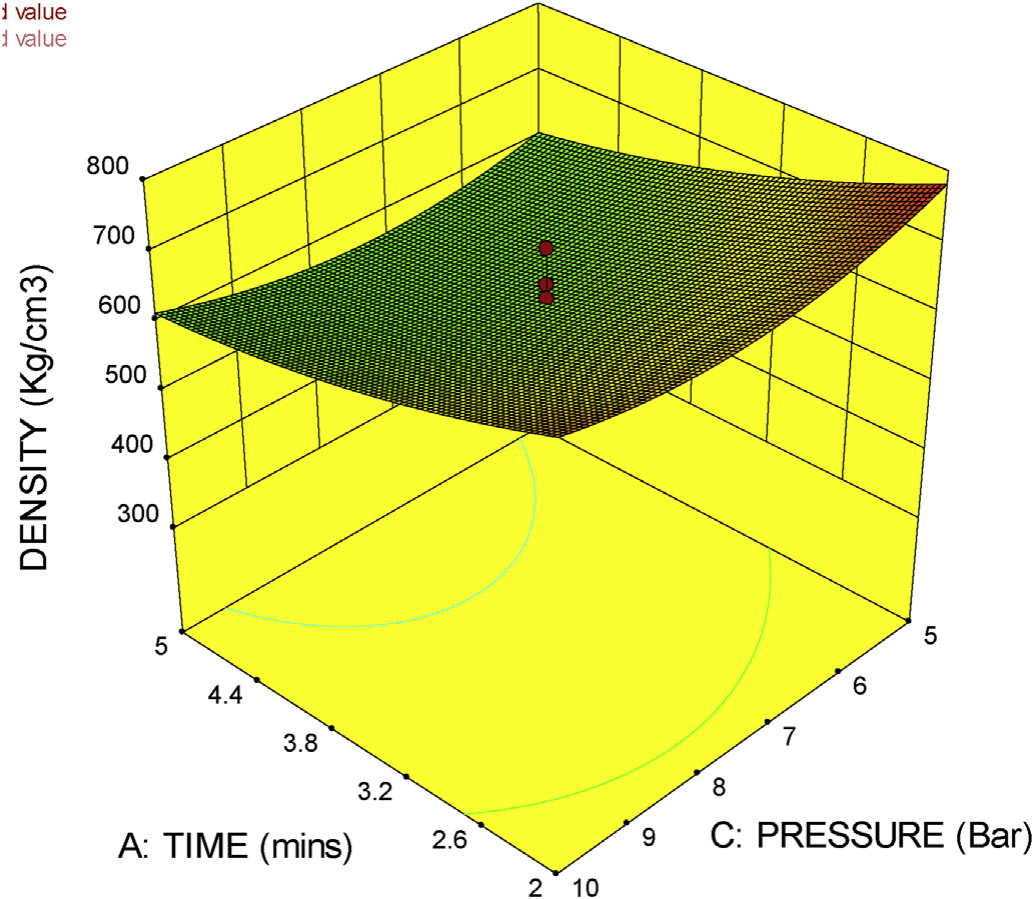
3D surface plot for press time against press pressure.

Figure 9 shows the 3D surface plot of press time against press pressure. It shows that an increase in press time and press pressure reduces the density of the material. This is true because if the press time increases the effect of pressure on the material will cause an increase in the amount of moisture content lost. This negative effect of press time and pressure was also reported by Ezenwa et al. (2019) in their research.

**Figure 10:**
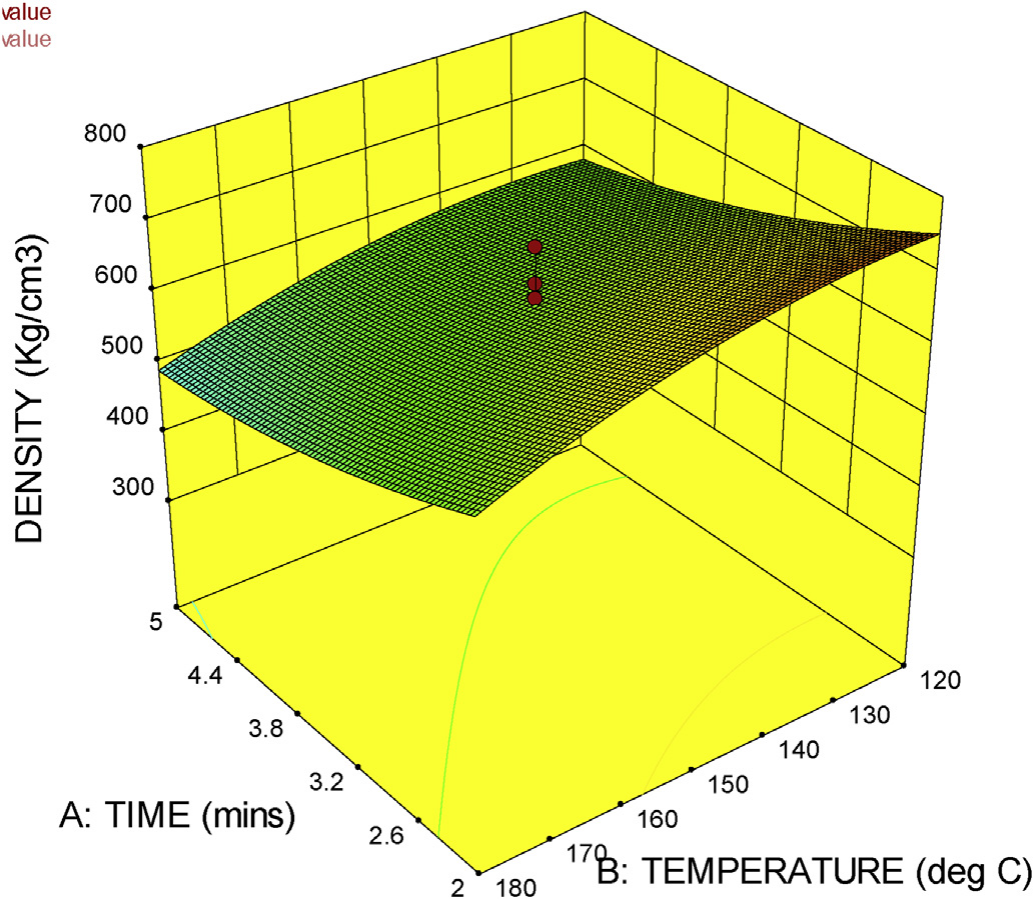
Figure 3D surface plot for press time against press temperature.

Figure 10, shows the 3D surface plot of press time against press temperature. The combined effect of press time and temperature can be seen from the figure to also have a negative effect on the density of the material, which is justified by the fact that both factors negatively affects the moisture content of the material.

The 3D surface plots in Figures 8, 9, and 10 gives a naturally justified combined effects of the factors (press time, press temperature and press pressure), on the response (density), thus the machine is proven to be in good working condition.

## 5 Conclusion

This work analysed the design of a constant temperature hydraulic press. The different parts of the equipment were designed using relevant mathematical expressions. In addition, the spring-mass analysis of the system was conducted to ensure proper functioning. As an advancement to previously existing designs, four columns with about 85KN tensile strength, were introduced to this design to ensure an even distribution of pressure on the press plate. The machine was designed to impact a maximum pressure of 25bar, using motorised hydraulic pump. On testing, the machine was used in the production of ceiling board using recycled Low Density Polyethylene (rLDPE) and the density of the developed material was studied against the press time, press temperature and press pressure over twenty experiments using the Design Expert software. The result gave 3D surface plots that validated the good working condition of the machine.

## Declarations

### Author contribution statement

Paul Chukwulozie Okolie, Echezona Nnaemeka Obika, Benjamin Segun Oluwadare, Obiora Nnaemeka Ezenwa & Chinedu Stanley Udensi: Conceived and designed the experiments; Performed the experiments; Analyzed and interpreted the data; Contributed reagents, materials, analysis tools or data; Wrote the paper.

### Funding statement

This research did not receive any specific grant from funding agencies in the public, commercial, or not-for-profit sectors.

### Competing interest statement

The authors declare no conflict of interest.

### Additional information

No additional information is available for this paper.
